# The bridge ventilator consortium – bringing trainees to the frontlines of innovation

**DOI:** 10.1080/10872981.2020.1826887

**Published:** 2020-09-30

**Authors:** Amir A. Hakimi, Aydin Zahedivash, Ellen M. Hong, Lily Y. Chen, Andrew E. Heidari

**Affiliations:** Chicago Medical School, Rosalind Franklin University of Medicine and Science, North Chicago, IL, USA; The University of Texas at Austin, Austin, TX, USA; Hackensack Meridian School of Medicine, Nutley, NJ, USA; College of Medicine, University of Central Florida, Orlando, FL, USA; Beckman Laser Institute and Medical Clinic, Irvine, CA, USA

**Keywords:** COVID-19, trainee, education, ventilator

Dear Editor,

The surge in Coronavirus Disease 2019 (COVID-19) cases leading to hospitalizations around the world has quickly depleted hospital resources and reserves. Based on observations in Wuhan, China and the Lombardy region in Italy, the real yet devastating possibility of too few Intensive Care Unit (ICU) beds and corresponding ventilator shortage in the USA was immediately recognized [1,2]. Faculty at the University of California – Irvine and University of Texas at Austin quickly acted to combat the expected ventilator shortage through the development of the Bridge Ventilator Consortium (BVC).

The BVC represents a multidisciplinary group that links industry, academia, government, non-profit organizations, and community members who have voluntarily partnered to develop breathing devices for use if ICU’s run short of conventional ventilators. Our designs transform readily available, consumer-grade products into basic transitory respiratory support devices. These transitional, or ‘bridge’ ventilators in turn increase availability of more powerful, intensive care capable ventilators for the most critical patients, and potentially expand surge capacity. Next to the possibility of an overwhelming medical supply shortage, the BVC has broadened its scope to develop non-invasive ventilation devices and personal protective equipment.

Our swift advancement from brainstorming design specifications to prototype development and testing can be attributed in large part to our grassroots in academic medicine. The COVID-19 pandemic demonstrated the unique ability of academic medical centers to interconnect faculty from distinct disciplines and mobilize the private sector that would have otherwise been siloed in their own industries. Within days, we were able to recruit a motivated team of physicians, engineers, scientists, legal advisors, respiratory therapists, and manufacturers among others to combat the pandemic.

Ambitious trainees have been at the forefront of each BVC operation, giving us firsthand exposure to the collaborative and innovative efforts that occur daily. School lectures have become supplemented with daily BVC meetings where we provide and receive real-time feedback on designs via teleconference. Laboratory closures have initiated the development of makeshift household workspaces to safely produce and evaluate prototypes. Extracurricular activities have been replaced with motor wiring tutorials and regulatory paperwork. Paradoxically, through these changes and efforts made at home, trainees have become more integrated in a global network.

In face of the new challenges brought on by COVID-19, trainees have stepped outside their comfort zones in medicine to explore and aid in engineering, manufacturing, and legal endeavors. The pandemic has indelibly changed medicine. As trainees, we will characterize this change through our growth as innovators, builders, collaborators, and leaders.
